# Efficacy and safety of canakinumab as a second line biologic after tocilizumab treatment failure in children with systemic juvenile idiopathic arthritis: A single-centre cohort study using routinely collected health data

**DOI:** 10.3389/fped.2023.1114207

**Published:** 2023-02-22

**Authors:** Ekaterina Alexeeva, Elizaveta Krekhova, Tatyana Dvoryakovskaya, Ksenia Isaeva, Aleksandra Chomakhidze, Evgeniya Chistyakova, Olga Lomakina, Rina Denisova, Anna Mamutova, Anna Fetisova, Marina Gautier, Dariya Vankova, Ivan Kriulin, Ruslan Saygitov

**Affiliations:** ^1^ Department of Rheumatology, National Medical Research Centre for Children's Health, Moscow, Russian Federation; ^2^ Department of Paediatrics and Paediatric Rheumatology, I.M. Sechenov First Moscow State Medical University (Sechenov University), Moscow, Russian Federation; ^3^ N.F. Filatov Clinical Institute for Children's Health, I.M. Sechenov First Moscow State Medical University (Sechenov University), Moscow, Russian Federation; ^4^ Association of Paediatric Rheumatologists, Moscow, Russian Federation

**Keywords:** systemic juvenile idiopathic arthritis, tocilizumab, canakinumab, second-line biologic, switching

## Abstract

**Background:**

A significant number of systemic juvenile idiopathic arthritis (sJIA) patients discontinue biologic disease-modifying antirheumatic drugs (bDMARDs) due to lack of efficacy or safety concerns. Studies of biologic therapy switch regimens in sJIA are required.

**Methods:**

Patients with sJIA who switched from tocilizumab (due to lack of efficacy or safety) to canakinumab (4 mg/kg every 4 weeks) and were hospitalized at the rheumatology department from August 2012 to July 2020 were included. Primary efficacy outcomes were 30% or greater improvement based on the paediatric criteria of the American College of Rheumatology (ACR30), achievement of inactive disease (JADAS-71 = 0) and clinical remission (ACR sJIA clinical inactive disease criteria). Follow-up from time first canakinumab dose administered was 12 months or the closest time point (not less than 6 and not more than 18 months). Data were extracted from electronic outpatient medical records.

**Results:**

During the study period, 46 patients with sJIA switched from tocilizumab to canakinumab. Median age at baseline was 8.2 [interquartile range (IQR) 4.0–12.9] years, with the median sJIA duration being 1.8 (IQR 0.8–5.8) years; 37 (80%) patients received at least one conventional DMARD (cDMARD; oral corticosteroids, methotrexate and/or cyclosporine A). Study outcomes were followed up in 45 patients (one patient did not attend the follow-up for an unknown reason); median follow-up was 359 (IQR 282–404) days. During the follow-up, 1 patient discontinued canakinumab due to tuberculosis detection and the dose was reduced or the injection interval increased in 4 (9%) patients. In total, 27 (60%) patients continued to receive at least one cDMARD. Improvement according to the ACR30 criteria was achieved in 43 patients [96%; 95% confidence interval (CI) 85–99], inactive disease in 42 (93%; 95%CI 82–98), and remission in 37 (82%; 95% CI 69-91); after adjustment for actual time-at-risk, the rates were 83, 85 and 73 events per 100 person-years, respectively. During follow-up, 23 AEs (most frequently infections) were reported in 19/45 (42%) patients; 5/45 (11%) patients developed macrophage activation syndrome, with a favorable outcome in all cases.

**Conclusions:**

One-year canakinumab therapy was found to be potentially effective as second-line biologic therapy after discontinuation of tocilizumab in patients with sJIA.

## 1. Background

The efficacy of interleukin-1 (IL-1) inhibitors (anakinra, canakinumab) and interleukin-6 (IL-6) inhibitors (tocilizumab) as first-line biologic therapy for children with systemic juvenile idiopathic arthritis (sJIA) has been practically assured. However, a significant number of patients with sJIA discontinue biologic therapy due to lack of efficacy or safety concerns (1–3). As a result, the patient must be switched to another biologic. Based on real-world observational studies, 21%–47% of patients with sJIA switch to a second, or subsequent, biologic disease-modifying antirheumatic drug (bDMARD) (1–3), within 7–16 months (2, 3). According to the Russian Register of patients with sJIA (2002–2015; *n* = 384), 68% of patients received at least one dose of at least one biologic (most often tocilizumab), with every third patient (32%) requiring a switch to a second or subsequent bDMARDs (4).

Current guidelines developed by American College of Rheumatology (ACR, 2013) (5) and National Health Service (NHS, 2015) (6) state that a biologic with a different mechanism of action should be prescribed when considering a second or subsequent biologic in patients with sJIA who have continued disease activity with, or an intolerance to, first-line biologic therapy. National Russian guidelines (2021) contain similar recommendations (7). To support this expert opinion, efficacy and safety studies of switch regimens in patients with sJIA are required (e.g., tocilizumab with a subsequent switch to canakinumab or any other dual regimen in line with current guidelines). In addition, when conducting such studies, it is important to consider the local/regional specificity of rheumatology care. Only two small-scale, open-label extension studies have demonstrated high efficacy of switching from tocilizumab to canakinumab in patients with sJIA at week 28 (8) [subsequently confirmed for 48 weeks (9)] and at month 12 (10).

This study aimed to evaluate the one-year efficacy and safety of canakinumab as a second-line biologic after tocilizumab treatment failure in children with sJIA in routine clinical practice.

## 2. Methods

### 2.1. Study design

This single-centre retrospective cohort study evaluated treatment outcomes in patients with sJIA who switched from tocilizumab to canakinumab. Data were collected for participants enrolled in one of two clinical trials at the study centre, CACZ885G2301E1 (G2301; NCT00891046) and CACZ885G2306 (G2306; NCT02296424) (11), in addition to those who switched treatment in routine clinical practice.

### 2.2. Setting

The patients were those who had been hospitalised and followed up at the rheumatology department of the National Medical Research Centre for Children's Health (previously known as the National Centre for Children's Health until November 2016 and then the National Scientific and Practical Centre for Children's Health, Moscow, until July 2017) in Moscow, from August 2012 (start of canakinumab studies at the centre) to July 2020 (uploading of the database with routinely collected health data to generate the study population).

### 2.3. Patients

Study included data of patients with sJIA aged 2 to <18 years who were prescribed to initiate canakinumab therapy over no more than 2 weeks after discontinuation of tocilizumab (received at least one dose of the drug) due to lack of efficacy or an adverse reaction were included in the analysis. The decision on the need to discontinue tocilizumab was made by the physician, as part of their routine clinical practice. Exclusion criteria not planned.

### 2.4. Treatment

All patients (both from routine practice and participants of G2301 and G2306) were prescribed canakinumab, a human anti-IL-1β monoclonal antibody, at a dose of 4 mg/kg subcutaneously every 4 weeks, maximum 300 mg. In accordance with the protocol, dose tapering was applied for participants of G2306 providing that they achieved clinical remission (inactive disease for ≥24 subsequent weeks) without the use of corticosteroids or methotrexate. Tapering regimens included either a dose reduction from 4 to 2 mg/kg (no earlier than Week 28), and then to 1 mg/kg (no earlier than Week 52), or increased inter-injection intervals, followed by discontinuation. Dose tapering was not scheduled for participants of G2301 or those in routine clinical practice. Any changes to therapy were made in accordance with the physician's decision.

### 2.5. Outcomes

Classification of primary and secondary outcomes were not pre-defined for patients switching to canakinumab in the clinical practice setting.

#### 2.5.1. Primary efficacy outcome

The study endpoints were as follows: at least 30% improvement based on the paediatric criteria of the American College of Rheumatology (ACR30) (12), the achievement of inactive disease according to the 71-point Juvenile Arthritis Disease Activity Score (JADAS-71 = 0 points) (13) and clinical remission according to the criteria of ACR sJIA clinical inactive disease (14). Follow-up for treatment outcomes was 12 months or the closest time, but not less than 6 and not more than 18 months from the day of the first dose of canakinumab.

#### 2.5.2. Secondary efficacy outcomes

##### 2.5.2.1. Clinical parameters

The percentage of patients with morning joint stiffness lasting for > 15-20 min, active joints, systemic signs of disease activity (fever, rash, serositis, hepatosplenomegaly and/or lymphadenopathy).

##### 2.5.2.2. Laboratory tests

Erythrocyte sedimentation rate (ESR) > 20 mm/h, C-reactive protein (CRP) levels > 5 mg/l.

##### 2.5.2.3. Assessment criteria

Non-zero values of disease activity as assessed by the treating physician using a 100-mm visual analogue scale (VAS), the patient's wellbeing assessed by the patient's parent using a 100-mm VAS and functional impairment based on the Childhood Health Assessment Questionnaire (CHAQ) (15) using the patient's parent estimate.

Monitoring of the treatment outcomes using additional parameters was conducted within the same time frame as for those using primary endpoints of the study.

#### 2.5.3. Safety outcomes

Safety of canakinumab-based therapy was assessed using the adverse event (AE) rates, i.e., any untoward medical events (unfavourable changes in the patient's health status or parameters reflecting the patient's health status) that occurred immediately after drug administration or during its use.

### 2.6. Data sources

Data on patients, administered treatment and its outcomes were extracted from electronic outpatient medical records of the rheumatology department and medical record documentation generated in local healthcare facilities during the time between hospital admissions (discharge summaries, outpatient medical records and medical reports). The latter were provided to the treating physicians of the rheumatology department by the parents of patients with sJIA. The analysis of laboratory parameters of the disease activity was carried out in the clinical diagnostic laboratory at the National Medical Research Centre of Children's Health. Health data were accumulated in the 1C:Enterprise application (1C Company, Russia). After uploading the routinely collected health data database (tables with data of all study subjects), the data entered were checked to exclude input errors.

Treatment efficacy measures (primary and secondary), as well as safety measures, were determined using the data contained in electronic outpatient medical records. When assessing treatment safety, information from medical record documentation provided by the patients' parents (cases of AEs in the between admission period) was also considered.

### 2.7. Statistical analysis

Statistical analysis was performed using SciStat, the online service by MedCalc Software (www.scistat.com). The description of quantitative values was presented as median (interquartile range, IQR). A Wilcoxon test was used to compare quantitative indicators in the dependent samples (before and after values). The event rates for the primary outcome measures were described indicating a 95% confidence interval (CI) without continuity correction (using online service www.vassarstats.net) and the rate per 100 person-years.

#### 2.7.1. Sensitivity analysis

Univariate Cox proportional hazards regression analysis was used to identify any predictors of clinical inactive disease achievement according to ACR sJIA criteria. The ACR30 and JADAS-71 criteria as such were abandoned due to the small number of patients with lack of efficacy. Treatment outcome were analysed taking into account the effect of all baseline clinical, laboratory, and assessment characteristics (at disease onset or before canakinumab treatment initiation).

### 2.8. Ethical approval

The protocols of G2301 and G2306 trials (dated 10 August 2012 and 5 December 2014, respectively) were approved by the local Ethics Committee of the National Medical Research Centre of Children's Health (at the time of the Ethics Committee opinion issue, referred to as National Centre for Children's Health). The inclusion of data from routine clinical practice in the study was not agreed with the Ethics Committee. At admission, the parents of all patients and patients aged ≥ 15 years provided their written informed consent allowing for the use of examination and treatment results for scientific purposes.

## 3. Results

### 3.1. Patient disposition

During the study period, a total of 250 patients with sJIA who received tocilizumab were monitored in the rheumatology department, of whom 65 (26%) discontinued therapy due to lack of efficacy or an adverse reaction. Tocilizumab was switched to canakinumab in 46 patients; 35 patients initiated canakinumab therapy in routine clinical practice, 3 upon entry to the G2301 trial and 8 upon entry to the G2306 trial. The most common reason for tocilizumab withdrawal was secondary treatment failure (defined by physicians as failure to meet the criteria of ACR sJIA clinical inactive disease occurring at any time following initial achievement of clinical remission following initial treatment effect; n = 32, 70%); 12 (26%) patients withdrew due to an adverse reaction, and 2 (4%) due to primary treatment failure (defined by physicians as failure to achieve ACR sJIA clinical inactive disease within the first 6 months after drug initiation).

The study outcomes were followed up in 45 patients, one patient did not attend the follow-up examination and treatment control in the period from month 6 to month 18, for an unknown reason.

### 3.2. Baseline characteristics

Patient demographics and disease characteristics are presented in Table 1. Approximately half of the patients (52%) were female. the median age at the time of sJIA onset was 3.4 years and median age at the time of canakinumab treatment initiation being 8.2 years. Duration of sJIA before canakinumab initiation did not exceed 2 years in more than half (52%) of the patients. Systemic manifestations (fever, rash, hepatomegaly/splenomegaly and/or lymphadenopathy) were observed in 42 (91%) patients. At the time of canakinumab initiation, all patients received treatment with non-steroidal anti-inflammatory drugs (NSAIDs), more than half received oral corticosteroids, more than received 40% methotrexate; and 17% received cyclosporine A. A total of 37 (80%) patients received at least one immunosuppressant (oral corticosteroids, methotrexate and/or cyclosporine A), of which 16 (43%) patients received concurrently ≥ 2 immunosuppressants.

**Table 1:** Patient characteristics at baseline (canakinumab treatment initiation) .

Variables	Values (*n* = 46)
General characteristics	
Female, *n* (%)	24 (52)
Age at baseline^a^, years	8.2 (4.0–12.9)
Age of disease onset, years	3.4 (2.4–5.5)
Duration of sJIA^a^, years	1.8 (0.8–5.8)
– <2 years (early arthritis), *n* (%)	24 (52)
Clinical characteristics at baseline^a^	
Systemic manifestations, *n* (%):	
– fever	35 (76)
– rash	33 (72)
– hepatomegaly/splenomegaly	26 (57)
– lymphadenopathy	17 (37)
– serositis (pleuritis, pericarditis)	6 (13)
Macrophage activation syndrome, *n* (%)	11 (24)
Active joint, *n* (%)	34 (74)
– active joint count (*n* = 34)	5 (2–8)
ESR, mm/h	26 (4–54)
– > 20 mm/h, *n* (%)	28 (61)
CRP, mg/l^b^	13 (1–62)
– > 5 mg/L, *n* (%)	31 (67)
Physician's assessment of disease activity, 0–100 mm VAS	45 (34–74)
Parent's assessment of overall well-being, 0–100 mm VAS	58 (45–81.5)
CHAQ score^c^	0.25 (0.03–0.88)
JADAS-71	15.5 (10–24)
Treatment at baseline	
NSAIDs, *n* (%)	46 (100)
Oral corticosteroids, *n* (%)	26 (57)
Methotrexate, *n* (%)	20 (43)
Cyclosporine A, *n* (%)	8 (17)

Quantitative variables are presented as median (IQR);.

^a^ On the day of canakinumab initiation.

^b^ CRP values below the analytical sensitivity threshold of the measurement method (< 1 mg/L) were coded as equal to 1 (in 13 patients).

^c^ Data from 12 (26%) patients with zero values of the parameter were disregarded. CHAQ, childhood health assessment questionnaire; CRP, C-reactive protein; ESR, erythrocyte sedimentation rate; JADAS, Juvenile Arthritis Disease Activity Score; sJIA, systemic juvenile idiopathic arthritis; VAS, visual analogue scale; NSAID, non-steroidal anti-inflammatory drug.

### 3.3. Primary efficacy outcomes

Median follow-up for patients with known outcomes (*n* = 45) was 359 (IQR 282–404) days; range, 184–476 days. Primary efficacy outcomes (paediatric ACR30 criteria, inactive disease based on JADAS-71, and clinical remission) were achieved in most of 45 patients in whom follow-up data was available (Table 2). After adjustment for actual time-at-risk, the rates were 83, 85, and 73 events per 100 person-years for ACR30, inactive disease, and clinical remission, respectively.

**Table 2:** Treatment outcomes after 12 months or the closest follow-up time (not less than 6 and not more than 18 months) after the first dose of canakinumab.

Variables	Values (*n* = 45)
Primary outcomes	
Paediatric ACR30, *n* [% (95% CI)]	43 [96 (85–99)]
JADAS–71, *n* [% (95% CI)]	42 [93 (82–98)]
Clinical remission, *n* [% (95% CI)]	37 [82 (69–91)]
Secondary outcomes	
Morning stiffness, *n* (%)	3 (7)
Active joints, *n* (%)	2 (4)
Systemic manifestation, *n* (%)	3 (7)^a^
ESR >20 mm/h, *n* (%)	4 (9)^b^
CRP >5 mg/L, *n* (%)	5 (11)
Physician's assessment of disease activity ≥0 by VAS, *n* (%)	8 (18)
Parents’ assessment of patient's wellbeing ≥0 by VAS, *n* (%)	8 (18)
CHAQ ≥0, *n* (%)	4 (9)

ACR, American College of Rheumatology; CHAQ, childhood health assessment questionnaire; CRP, C-reactive protein; ESR, erythrocyte sedimentation rate; JADAS, Juvenile Arthritis Disease Activity Score; sJIA, systemic juvenile idiopathic arthritis; VAS, visual analogue scale.

^a^ disease manifestations with fever in 2 patients, hepatomegaly/splenomegaly and lymphadenopathy in 1 patient.

^b^ *n* = 44.

Abovementioned outcomes were achieved with some treatment modifications. In particular, during follow-up, canakinumab was discontinued in 1 patient [discontinuation occurring after 14 months due to tuberculosis (TB) detection], the dose was reduced to 2 mg/kg per injection in 3 patients, and the interval between injections was increased up to 8 weeks in 1 patient. In addition, during the follow-up period, NSAIDs were administered in 2 (4%) patients, oral corticosteroids in 20 (44%), methotrexate in 12 (27%) and cyclosporine A in 9 (20%) patients. A total of 27 (60%) patients received at least one immunosuppressant (oral corticosteroids, methotrexate and/or cyclosporine A), of which 13 (48%) patients received concurrently ≥ 2 immunosuppressants.

### 3.4. Secondary efficacy outcomes

At the time of the follow-up examination, some signs of disease persisted in 4%–18% of patients (Table 2). At least one clinical sign of the active disease (morning stiffness, active joints, systemic disease manifestations) was noted in 7 of 45 (16%) patients. At the end of follow-up, median ESR was 3 (IQR 2–7) mm/h (data presented for 44 patients) (*p* < 0.001; hereinafter vs. the baseline), median CRP (*n* = 45) was 1.0 (IQR 1.0–1.9) mg/l (*p* < 0.001). At least one laboratory sign of the disease was noted in 6/45 (13%) patients. Nonzero scores for at least one estimated parameter of disease activity (activity assessment by the physician, parents' assessment of patient's wellbeing, functional insufficiency according to CHAQ) were identified in 8/45 (18%) patients.

In total, at the end of follow-up, any of the above clinical, laboratory and/or estimated parameters of disease activity was observed in 11 (24%) of 45 patients, of which 5 had only clinical, laboratory or estimated sign of active disease.

### 3.5. Safety

During follow-up, 23 AEs were reported in 19/45 (42%) patients, of whom 11 had an acute respiratory or intestinal infection, 4 had leukopenia/neutropenia, 4 had elevated liver transaminase levels and 4 had TB infection (including one case of a positive *M. tuberculosis* test result). Electronic medical records of 5/45 (11%) patients contain the history of macrophage activation syndrome (MAS) during the use of canakinumab, with a favourable outcome in all patients.

### 3.6. Predictors of remission

According to the criteria of ACR sJIA clinical inactive disease, none of the sample characteristics (see Table 1) were associated with achievement of clinical remission during follow-up.

## 4. Discussion

### 4.1. Summary of primary findings

Most sJIA patients treated with canakinumab after discontinuation of tocilizumab (74% due to lack of efficacy, either primary or secondary) achieved remission (82%), inactive disease (93%) and/or improvements in disease signs (ACR30 – 96%) within a year. In addition, the use of c DMARDs, such as oral corticosteroids, methotrexate and/or cyclosporine A, also decreased. The “survival” of canakinumab therapy during follow-up was high, with only one case of therapy withdrawal due to TB infection. At least one AE (most frequently infection) was reported in more than 40% of patients.

### 4.2. Study limitations

#### 4.2.1. Representativeness of the study sample

The study involved patients who were followed up at a single centre only. In these cases, the patient management does not reflect all specific aspects of routine clinical practice in the study region and the extent to which these findings can be generalised to other healthcare systems is unknown. Moreover, the rheumatology department, whose patients were enrolled in the study, is a subdivision of the largest Russian paediatric centre, the capabilities of which (expert, diagnostic, therapeutic) can significantly exceed those of regional clinical centres. This is reflected by the enrolment of every fourth patient (*n* = 11; 24%) we analysed in the international clinical trial (G2301 and G2306). In these trials, therapy is pre-determined by the protocol and treatment control and disease outcomes monitoring are more stringent than in routine clinical practice. In addition, this study enrolled patients from the Russian Federation only; it is not known to what extent the findings can be generalised to patients in other countries with other healthcare systems.

#### 4.2.2. Small-scale sample

One of the important advantages of large-scale cohort studies is the heterogeneity of its sample, helping to gain insight into the results of medical interventions in a setting as close to real-world clinical practice as possible. In small-scale trials, the description of relatively rare treatment outcomes (both favourable and unfavourable, including AEs) is obviously impossible.

#### 4.2.3. Variability of follow-up period

Many of our paediatric patients were from remote regions of Russia, which limited their ability to attend regular follow-ups. Attending the clinical centre for at least one assessment per year was recommended for all patients, but some were unable to do this routinely. As a result, the spread of the actual follow-up values varied from month 6 to month 16. Although the median period was 12 months, the distribution of the follow-up duration in the study sample was shifted to the left (coefficient of skewness −0.271). This limits the interpretation of the study results.

#### 4.2.4. Routinely collected health data

We analysed the data obtained from electronic health records. The quality of such data may give rise to reasonable doubts (16), particularly with regards to safety information. Furthermore, missing data is often a limitation for the use of medical records. In this study, there were only isolated cases of missing data, which, in our opinion, did not significantly affect the results obtained.

#### 4.2.5. Absence of control group

We cannot directly attribute the effects of therapy (both beneficial and adverse) solely to the effect of canakinumab, since most patients (80% at baseline and 60% at follow-up) received at least one more immunosuppressant agent, such as oral corticosteroids, methotrexate and/or cyclosporine A. Similarly, it should be noted that approximately three-quarters of patients were prescribed canakinumab in view of lack of efficacy of tocilizumab (mainly secondary failures) and at the same time, while on canakinumab, a decrease in the proportion of patients receiving oral corticosteroids (from 57% to 44%) and methotrexate (from 43% up to 27%) was reported. The results described with canakinumab treatment were therefore achieved against a less intense background regimen of cDMARDs. Moreover, this does not take into account a change in the dose of cDMARDs, the reduction of which has been shown in randomised clinical trials with canakinumab (9, 17).

### 4.3. Interpretation of study results

#### 4.3.1. Efficacy of switching from tocilizumab to canakinumab

In our study, the minimum significant improvement according to the paediatric ACR30 criteria was reported in 96% (43/45) of patients (median follow-up of 12 months or 359 days) or in 85% in terms of the rate per 100 person-years. Similar results were demonstrated in clinical trials by Brunner et al., 2015 (10) and Nishimura et al., 2021 (9). In the former study, after switching from tocilizumab (withdrawn due to lack of efficacy or safety) to canakinumab, the ACR30 criteria was achieved in 23/24 (96%) patients with sJIA at month 12, in the latter, in 16/19 (84%) at approx. month 11 (median 337 days). A similar result (ACR30 in 86% at the median follow-up of 8 months or 236 days) was obtained in a clinical trial (withdrawal phase) by Ruperto N. et al., 2012, where 80% of patients received tocilizumab before canakinumab (17). Moreover, in the above-mentioned trials, roughly one third of patients discontinued glucocorticoids following initiation of canakinumab (9, 17); in our study, oral glucocorticoids were discontinued in 6 of the 26 patients (23%) who were receiving these drugs at the time of administration of the first dose of canakinumab.

Several hypotheses can explain the lack of efficacy of tocilizumab in patients with sJIA and, accordingly, the efficacy of switching to canakinumab. First, IL-1 but not Il-6, can be considered a primary mediator of autoimmune (in fact, aseptic) inflammation. In particular, *in vitro* studies demonstrate that sJIA is an IL-1*β*-mediated disease (18). The pathogenetic role of these cytokines in the development of autoimmune processes and arthritis was confirmed both in IL-1Ra (19) and IL-6-deficient mice (20) as well as the initial clinical studies of IL-1- (18) and IL-6-inhibitors (21). However, it is noteworthy that systemic inflammation in combination with arthritis is observed in IL-1-overexpressing (22), but not IL-6-overexpressing transgenic mice (23, 24). In addition, the aseptic inflammation model showed that IL-6 is not detectable in the blood serum of IL-1β-deficient mice (25), while, on the contrary, IL-1 is induced normally in IL-6-deficient mice (26). It was also found that in mice transgenic for IL-1*α* (activation of IL-1 signalling) and prone to arthritis, IL-6 deficiency resulting from knockout of the corresponding gene reduces, but does not cancel, pathological changes in the joints (22). Finally, in newly diagnosed untreated patients with sJIA, high expression levels of genes involved in the negative regulation of IL-1R signalling have been observed (27).

Secondly, different sensitivity to tocilizumab in patients with sJIA may be fundamental. For instance, it has been shown that the lack of efficacy of tocilizumab is associated with initially high concentrations of IL-6 (28, 29), sIL-6R (30, 31) and a low concentration of soluble gp130 (29), which is a natural inhibitor of the IL-6 trans-signalling pathway (32). The effect of high levels of IL-6 may be due to the competition of significant amounts of cytokine with tocilizumab for binding sites (IL-6R) on the membranes of target cells, i.e., immunocompetent cells and hepatocytes, but not for soluble forms of the receptor, which is confirmed in studies with IL-6-dependent myeloma cell line and recombinant BaF/IL-6R (33). It can be assumed that in some patients the recommended dose of tocilizumab may not be enough to suppress both a high concentration of soluble IL-6R and significant amounts of the IL-6/sIL-6R complex due to a low concentration of soluble gp130.

Thirdly, there are rare cases of treatment failure and/or hypersensitivity reactions associated with antibodies to biologics and, in particular to tocilizumab (34). In these cases, the efficacy of IL-1 inhibitors can be considered as a therapy for “biologically naïve” patients. The development of antibodies to canakinumab in such patients is unlikely as no anti-drug neutralising antibodies were detected (11, 35).

Finally, tocilizumab does not affect the concentration of IL-1β (28), i.e., does not have an indirect effect on the key mechanism of sJIA pathogenesis. In this regard, it is important to note that high baseline levels of IL-1 in serum is associated with a lower efficacy of tocilizumab after 16 (29) and 52 weeks (36) of therapy. In contrast, down-modulation of IL-6 has been reported as a result of therapy with IL-1 inhibitors, occurring as early as 1 week after anakinra initiation (37) and 3 days after the administration of canakinumab (38). Down-modulation of IL-6 in patients with sJIA on canakinumab therapy (switching from tocilizumab) has been shown to persist for 24 and 48 weeks (9).

Therefore, the benefits of switching from an IL-6 inhibitor to an IL-1 inhibitor and, in particular from tocilizumab to canakinumab can potentially be explained by: IL-1-mediated links in the pathogenesis of sJIA; unequal sensitivity to tocilizumab in patients and the effect exerted by IL-1 inhibitors on the sJIA mechanisms associated both with IL-1 and IL-6.

#### 4.3.2. Safety of switching from tocilizumab to canakinumab

In our study, AEs were reported in more than 40% of patients. According to the findings from clinical studies in patients with sJIA who received canakinumab after tocilizumab, AEs were reported in 78%–100% of patients (9, 17). This difference can be explained by the study design and, in particular, using medical records to estimate the AEs. There is no doubt that routinely collected health data include only the most remarkable deviations in the health status, which, in the physicians' opinion, are obviously related to the prognosis. In this regard, our data on the frequency of AEs are consistent with the rates of serious AEs reported in the study by Nishimura K. et al. (42%) (9). According to the German BiKeR Registry, the rate of serious adverse events with canakinumab was even lower, 20 cases per 100 patient-years (39).

It should be separately noted that in our study, 11% of patients developed MAS while using canakinumab and that this is higher than the rate observed in clinical trials of canakinumab (5.3% or 2.8 per 100 patient-years) (40) where the incidence rate of (probable) MAS observed in canakinumab-treated patients was comparable to the incidence reported in patients in the placebo group. In part, the higher incidence of MAS in our study may be due to the limited diagnostic accuracy of the criteria for this condition (over-diagnosis) (41). Indeed, in small-scale studies [with < 100 subjects (27, 42, 43)] the frequency of reported cases of MAS significantly exceeds that reported in large-scale studies [> 100 patients (44–46)] (12%–20% vs. 4%–7%, respectively).

## 5. Conclusion

One-year canakinumab therapy was found to be potentially effective as second-line biologic therapy after discontinuation of tocilizumab in patients with sJIA. Efficient treatment with canakinumab was achieved along with a decrease in the use of cDMARDs, such as oral corticosteroids and methotrexate. The “survival” of canakinumab therapy during the follow-up was high, with only one case of drug withdrawal. At least one AE (most frequently – infections) was reported in more than 40% of patients. Long-term data needs to be evaluated to see the benefit of canakinumab in patients with sJIA over a longer period.

## Data Availability

The datasets presented in this article are not readily available because Open study data sharing is not applicable due to local regulations. Requests to access the datasets should be directed to Ekaterina Alexeeva, alekatya@yandex.ru.
